# SALL4 correlates with proliferation, metastasis, and poor prognosis in prostate cancer by affecting MAPK pathway

**DOI:** 10.1002/cam4.5998

**Published:** 2023-04-29

**Authors:** Jie Zhou, Shengmeng Peng, Huiyang Fan, Jin Li, Zean Li, Ganping Wang, Lexiang Zeng, Zhenghui Guo, Yiming Lai, Hai Huang

**Affiliations:** ^1^ Department of Urology, Sun Yat‐sen Memorial Hospital Sun Yat‐sen University Guangzhou China; ^2^ Guangdong Provincial Key Laboratory of Malignant Tumor Epigenetics and Gene Regulation, Sun Yat‐sen Memorial Hospital Sun Yat‐sen University Guangzhou China; ^3^ Guangdong Provincial Clinical Research Center for Urological Diseases Guangzhou China; ^4^ Department of Reproductive Center, Sun Yat‐sen Memorial Hospital Sun Yat‐sen University Guangzhou China; ^5^ Department of Urology, Zhujiang Hospital Southern Medical University Guangzhou China; ^6^ Department of Urology The Sixth Affiliated Hospital of Guangzhou Medical University, Qingyuan People's Hospital Qingyuan China

**Keywords:** MAPK pathway, metastasis, prostate cancer, SALL4

## Abstract

**Background:**

The mechanism involved in prostate cancer (PCa) metastasis is still poorly understood, and several oncogenes are known to regulate this process. However, the role of spalt‐like transcription factor 4 (SALL4) in PCa metastasis remains unclear.

**Methods:**

We performed RNA‐sequencing to compare the mRNA expression profiles of seven localized PCa tissues and six metastatic PCa tissues. SALL4 was then identified and compared in the localized PCa and metastatic PCa. Immunohistochemical studies, qRT‐PCR, and Western blot were performed to analyze the expression of SALL4 in PCa patients and cell lines. SALL4 expression and its relevance to clinical traits and prognosis were further explored in the TCGA database and in our 68 clinical samples. Subsequently, we knocked down SALL4 in DU145 and PC3 cells and performed a series of functional assays to explore the effect of SALL4 on PCa progression. Finally, protein levels of SALL4 and core components of the MAPK pathway were measured by Western blot, and cells were treated with PD0325901 to observe proliferation and metastasis.

**Results:**

Significantly higher expression of SALL4 was found in metastatic PCa than in localized PCa. In addition, high SALL4 expression was significantly associated with high pathological T stage, N stage, Gleason score, and poor disease‐free survival in TCGA database and in our clinical samples. Functional studies indicated that knockdown of SALL4 in DU145 and PC3 inhibited proliferation, migration, and angiogenesis. Furthermore, the ERK and P38 protein phosphorylation significantly reduced after knockdown of SALL4 in DU145 and PC3, indicating the inactivation of the MAPK signaling pathway. Finally, the proliferation and migration ability of DU145 and PC3 cells were significantly decreased after PD0325901 treatment.

**Conclusions:**

SALL4 predicts unfavorable outcome and is closely associated with PCa progression, suggesting that SALL4 may be a promising prognostic marker and potential therapeutic target for PCa.

## 1 INTRODUCTION

Prostate cancer (PCa) is one of the most common cancers in men worldwide, with 268,490 new cases to be diagnosed in the United States in 2022.^1^ A significant proportion of patients miss the opportunity for radical surgery because PCa develops insidiously in its early stages, leading to progression to advanced stages. The 5‐year survival rate has been reported to be only 31% for patients with metastatic PCa (mPCa).^1^ Globally, there are more than 350,000 deaths related to PCa each year, making it a leading cause of cancer‐related deaths in men.^2^ Therefore, identifying specific biomarkers and more effective therapeutic targets for metastatic PCa is imperative.

Spalt‐like transcription factor 4 (SALL4) is a transcription factor first described in *Drosophila*, and plays a critical role in maintaining stemness and fetal cell renewal capacity through a variety of potential mechanisms.^3^, ^4^ It has been reported that SALL4 promotes stem cell self‐renewal and inhibits stem cell differentiation by adhering to β‐catenin and participating in the regulation of the Wnt/β‐catenin pathway.^5^ Another study has shown that key stemness factors, such as OCT4 and NANOG, are regulated by SALL4.^6^, ^7^

In addition to the above aspects of research, SALL4 has been increasingly studied in tumor development in recent years.^8^ Although SALL4 protein expression is barely detectable in mature tissues, it is reactivated in some human tumors. Elevated expression of SALL4 has been observed in several cancers, including acute myeloid leukemia, germ cell tumors, gastric cancer, lung cancer, ovarian cancer, and glioma.^5^, ^8^, ^9^, ^10^, ^11^ SALL4 overexpression can promote cancer proliferation, migration, invasion, and progression.^8^ It has been found that SALL4 promotes gastric cancer metastasis by inducing epithelial–mesenchymal transition.^10^ SALL4 also inhibits apoptosis by binding to the promoters of genes involved in apoptosis. Some studies have shown that SALL4 regulates apoptosis by affecting BCL‐2 on the apoptotic pathway in leukemia patients. Similar findings have been found in breast cancer.^12^, ^13^ SALL4 knockdown in cancer cells also induces cell cycle arrest at G1.^14^, ^15^ All these studies suggest that SALL4 serves an important role in tumor progression. However, the functions and mechanisms of SALL4 in PCa remain largely unknown and, therefore, further studies are needed to elucidate.

In our study, we performed RNA sequencing and found that SALL4 was significantly more highly expressed in mPCa than in localized PCa. This was confirmed by cell lines and clinical samples, and then we investigated the correlations between SALL4 expression and clinical features and prognosis. Furthermore, we explored the effect of knocking down SALL4 on the proliferation, apoptosis, migration, and angiogenesis of PCa cell lines and its potential mechanisms in vitro experiments. In conclusion, our data demonstrate that SALL4 is associated with proliferation, metastasis, and poor prognosis of PCa and that the MAPK signaling pathway is involved, implying that SALL4 can serve as a prognostic marker and potential therapeutic target for PCa.

## 2 MATERIALS AND METHODS

### 2.1 RNA sequencing (RNA‐seq)

We collected a total of 13 samples for RNA‐seq, including seven cases of PCa primary sites without metastasis and six cases of primary sites of bone metastatic PCa, that underwent surgery or biopsy from Sun Yat‐sen Memorial Hospital of Sun Yat‐sen University (Guangzhou, China). The clinicopathological characteristics of the 13 patients are summarized in Table S1. Total RNA was extracted from tissues using the Trizol (invitrogen) according to the manufacturer's protocol, and ribosomal RNA was removed using the Ribo‐Zero™ kit (Epicentre, Madison, WI, USA). Fragmented RNA (the average length was approximately 200 bp) were subjected to first strand and second strand cDNA synthesis following by adaptor ligation and enrichment with a low‐cycle according to instructions of NEBNext®Ultra™ RNA Library Prep Kit for Illumina (NEB, USA). The purified library products were evaluated using the Agilent 2200 TapeStation and Qubit®2.0 (Life Technologies, USA). The libraries were paired‐end sequenced (PE150, Sequencing reads were 150 bp) at Guangzhou RiboBio Co., Ltd. (Guangzhou, China) using IlluminaHiSeq 3000 platform. All raw data of RNA‐seq have been uploaded to National Genomics Data Center (https://ngdc.cncb.ac.cn/).

### 2.2 GEO and TCGA database mining

GSE3325 dataset and GSE6752 dataset are available at Gene Expression Omnibus (GEO) (https://www.ncbi.nlm.nih.gov/geo/). GSE3325 dataset, including seven localized PCa samples and six mPCa samples, and GSE6752 dataset, including 10 primary prostate tumors and 21 prostate tumor metastases, were analyzed with the GEO2R tool. Only genes with log2 fold change ≥1 and adjust *p* value <0.05 were selected for further study. The TCGA‐PRAD dataset including 499 PCa samples and 52 adjacent normal prostate tissue samples was obtained as previously described.^16^

### 2.3 Clinical prostate cancer samples

Sixty‐eight PCa samples were collected. These prostate tissue samples from biopsy or surgery had been donated to be used in the current study by patients from 2005 to 2014 at Sun Yat‐Sen Memorial Hospital. All of the samples were formalin‐fixed and paraffin‐embedded.

### 2.4 Immunohistochemistry (IHC)

IHC was performed as previously described.^17^ Briefly, the percentage of positive staining (SALL4 frequency) was scored as follows: 0 for no cells with positive staining; 1 for 1%–25% of cells stained; 2 for 26%–50% of cells stained; 3 for 51%–75% of cells stained; and 4 for >75% cells stained. The staining intensity (SALL4 intensity) was scored as 0 for not stained, 1 for weakly stained, 2 for moderately stained, and 3 for strongly stained. Both the frequency and intensity of SALL4 staining were assessed in a double‐blind manner. The SALL4 level scores were the sums of the scores of SALL4 frequency and intensity. Finally, the SALL4 expression was classified as low (score 0–2), moderate (score 3–4), or high (score 5–7).

### 2.5 Cell cultures and transfections

PCa cell lines (DU145, PC3, LNCaP, C42, and 22Rv1) and normal prostate‐derived cell line (RWPE‐1) were obtained from American Type Culture Collection (ATCC, USA) and the cell bank Center of Experimental Animal Sun Yat‐sen University (Guangzhou, China). Cells were cultured in RPMI‐1640 (HyClone, USA) medium supplemented with 10% fetal bovine serum (Biological Industries, Israel), while the RWPE‐1 was cultured in Keratinocyte serum‐free medium (Invitrogen, USA) supplemented with epidermal growth factor and bovine pituitary factor. All cells were maintained in a 37°C, 5% CO_2_ incubator. Transfections were conducted with Lipofectamine RNAiMAX transfection reagent (Thermo Fisher Scientific, USA) in accordance with the manufacturer's instructions. All siRNAs are shown in the Table S2. Cell cycle assay, qRT‐PCR, and migration assay, etc. were performed 48 h after transfection. Extraction of proteins for WB was performed at 72 h.

### 2.6 Quantitative reverse transcriptase‐PCR (qRT‐PCR)

RNA‐Quick Purification Kit (ESscience, China) was used to extract total RNA. 500 ng of total RNA was used for Reverse transcriptase reaction (RT) using HiScript III All‐in‐one RT SuperMix Perfect for qPCR (Vazyme, China). The expression of mRNA in cell lines was quantified using the ABI QuantStudio Sequence Detection System (Applied Biosystems). Expression levels were normalized by GAPDH. All the primers used in the present study are listed in Table S3.

### 2.7 Western blot (WB)

Proteins in cell samples were harvested using RIPA lysis buffer (Beyotime, China) supplemented with protease inhibitors and phosphatase inhibitors. WB was performed following the previously described method.^17^ Table S4 lists the primary antibodies used in this study.

### 2.8 Cell viability assay and flow cytometry analysis

PCa cells already treated accordingly were cultured in 96‐well plates. Using the MTS assay, cell viability was quantified by measuring absorbance using a Multiskan MK3 microplate reader (Thermo Fisher, USA). The percentage of apoptotic cells was assayed by the previously reported method.^18^

### 2.9 Cell cycle assay

Cell Cycle Detection Kit (KeyGEN BioTECH, China) was used to detect cell cycle. 5 × 10^5^ harvested cells were washed with PBS and then fixed in 70% prechilled ethanol at 4°C overnight. The specific steps are all in accordance with the manufacturer's instructions. Cell cycle assay was performed using the CytExpert flow cytometer (Beckman Coulter, Inc.) .1 × 10^4^ events were measured in the flow cytometer analysis. ModFit software (BD) was used to analyze the results.

### 2.10 Transwell migration assay

Migration assay was performed 48 h after transient transfection. The bottom of each well of the 24‐well plate culture dish (Corning, USA) was filled with DMEM or RPMI 1640 medium containing 10% FBS. The chambers were covered with polyethylene terephthalate (PET) membranes with 8‐μm pores, and 50,000 cells/well in serum‐free DMEM or RPMI 1640 were added to the top culture insert. The next steps were conducted as described previously.^17^

### 2.11 Tube formation assay

DU145 and PC3 treated with negative control siRNA or SALL4 siRNA were cultured as described above. When the cells reached 70%–80% confluence, the serum‐free medium was replaced, and the supernatant was collected after 24 h. Thawed Matrigel at 4°C overnight, added 50 μL Matrigel uniformly into 96‐well plate and then incubated at 37°C for 1 h. The logarithmic growth phase human umbilical vein endothelial cells (HUVECs) were resuspended in the collected supernatant to a concentration of 2 × 10^5^/mL, added 100 μL suspension to the 96‐well plate and cultured for 5–12 h. Took out the 96‐well plate and randomly selected three fields of view under the inverted microscope to take tube images and analyzed with Image‐Pro Plus (IPP) software.

### 2.12 PD0325901 assay

DU145 and PC3 treated with negative control or PD0325901 (10 μM) (a well‐known MEK/ERK pathway inhibitor) were incubated for 48 h as described above, followed by colony formation assay and transwell migration assay. Thousand cells were used for colony formation, and the colonies were counted after 14 days (AID vSot Spectrum, Germany).

### 2.13 Statistical analysis

All quantitative data are based on three independent experiments and presented as the mean ± SD. Statistical analyses were conducted using SPSS version 23.0 software and R package (v4.1.1), and graphs were generated using GraphPad Prism 9 software and R package. The Wilcoxon rank‐sum test or two independent sample *t*‐test was used to compare the expression of SALL4 between two groups. The association between SALL4 expression level and clinical features in TCGA‐PRAD was analyzed using chi‐squared test, two independent sample t‐test, or Kruskal–Wallis test. Correlation analysis of immunohistochemical staining with clinicopathological parameters using nonparametric tests. *p* < 0.05 was considered significantly.

## 3 RESULTS

### 3.1 Identification of SALL4 as an upregulated gene closely associated with PCa metastasis

**FIGURE 1:**
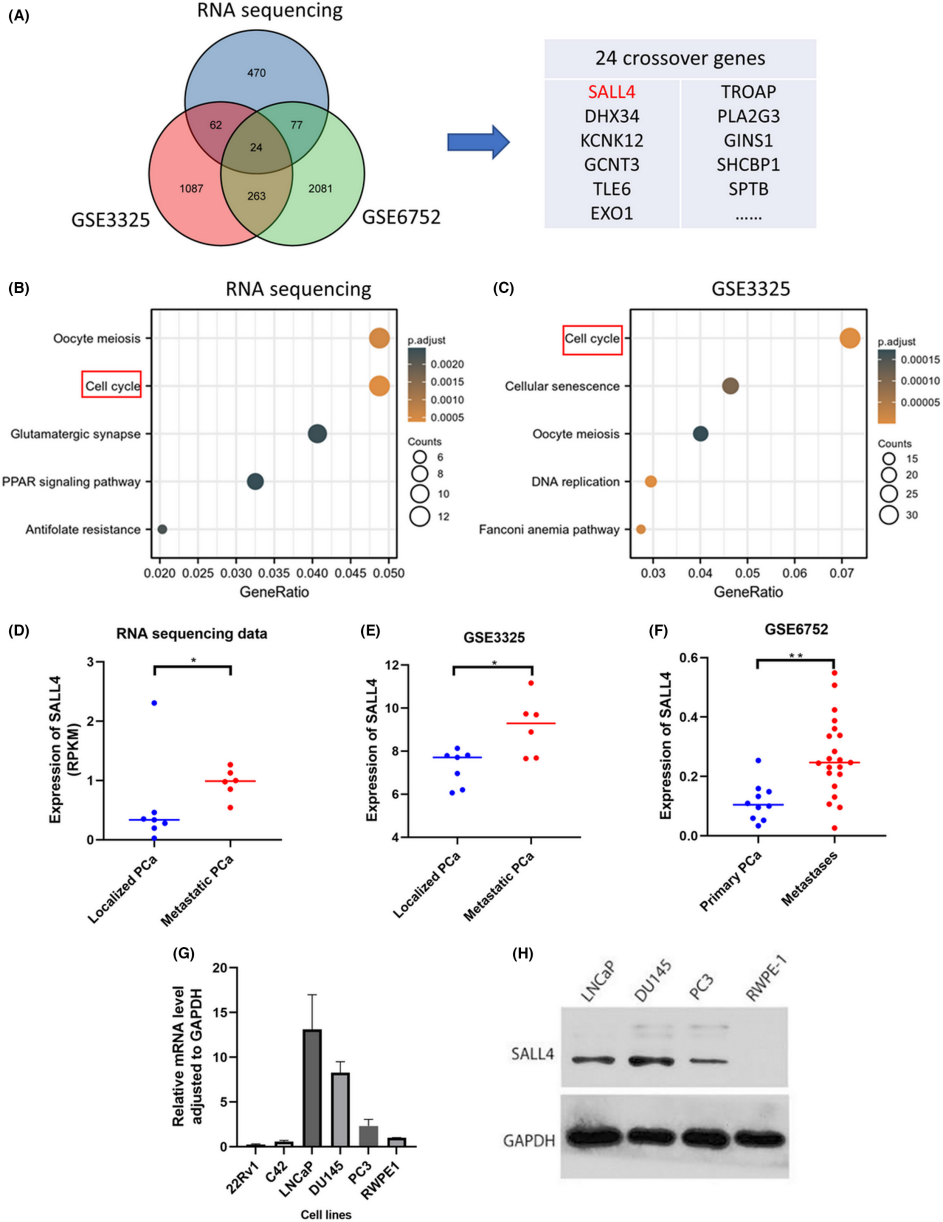
Identification of SALL4 as an upregulated gene closely associated with PCa metastasis. (A) High expression of SALL4 in metastatic PCa confirmed by our RNA sequencing (RNA‐seq) and two GEO datesets (including GSE3325 and GSE6752). (B) Enrichment analysis of 633 upregulated genes in our RNA‐seq data showed that the top‐ranked pathways were oocyte meiosis and cell cycle. (C) Enrichment analysis of 1436 upregulated genes in the GSE3325 dataset also revealed that the top‐ranked pathways were cell cycle, oocyte meiosis, and DNA replication. (D) Comparison of SALL4 expression in localized PCa and metastatic PCa in our RNA‐seq data. (E,F) SALL4 expression in GSE3325 dataset and GSE6752 dataset. (G) SALL4 mRNA levels in prostate cancer cells and human prostate epithelial cells RWPE‐1. (H) SALL4 protein levels in LNCaP, DU145, PC3, and RWPE‐1 cell lines were detected by Western blot. *p < 0.05; **p < 0.01; ns *p* > 0.05.

To study PCa metastasis‐associated genes, as described above, we first performed RNA‐seq. Compared with localized PCa, a total of 633 differentially expressed protein‐coding genes with log2 fold change ≥1 and adjusted *p* value <0.05 were identified in mPCa. Then, 1436 and 2445 genes associated with PCa metastasis were identified in the GSE3325 and GSE6752 datasets, respectively. Next, by taking the intersection, we obtained 24 crossover genes for subsequent study, including SALL4, SRRM2, DHX34, KCNK12, GCNT3, PLCXD1, CEL, TLE6, CBX2, FOXM1, NUP210, RAD54L, PITPNM2, CCNE2, SLCO4A1, ESPL1, MMP8, MGAM, TROAP, PLA2G3, GINS1, SHCBP1, SPTB, and EXO1 (Figure 1A). We then used the GEPIA2 website (http://gepia2.cancer‐pku.cn/#survival) to analyze the association of these 24 genes with disease‐free survival (DFS) in prostate cancer. Among these prognosis‐associated genes, by searching PUBMED website we excluded genes (CBX2, FOXM1, CCNE2, ESPL1, TROAP, and EXO1) that have been studied in prostate cancer.

To investigate which biological processes/pathways play an important role in PCa progression, we performed enrichment analysis. Enrichment analysis of 633 metastasis‐associated upregulated genes identified by our RNA‐seq revealed that the top‐ranked pathways were oocyte meiosis and cell cycle (Figure 1B). In addition, enrichment analysis of 1436 upregulated genes in the GSE3325 dataset also indicated that the top‐ranked pathways were cell cycle, oocyte meiosis, and DNA replication (Figure 1C). Therefore, we have reason to believe that cell cycle‐related genes and pathways play a critical role in PCa progression. Among these remaining genes, SRRM2, PLCXD1, DHX34, KCNK12, TLE6, and others have been rarely reported to promote tumor proliferation. It was previously documented that SALL4 is a transcription factor that can influence the cell cycle and promote tumor proliferation and metastasis.^14^, ^15^, ^19^ More importantly SALL4 has not been well studied in PCa.

As mentioned above, SALL4 was identified as an upregulated gene closely associated with PCa progression. High expression of SALL4 was found in our RNA‐seq data and GSE3325 dataset, and the difference was statistically significant (Figure 1D,E). Furthermore, SALL4 expression was found to be elevated in PCa metastases compared to primary PCa in the GSE6752 dataset (Figure 1F). To further determine the expression of SALL4, we analyzed the difference of SALL4 expression between PCa cell lines and normal human prostate epithelial RWPE‐1 by qRT‐PCR and Western blot. As shown in Figure 1G and Figure 1H, the mRNA and protein expression of SALL4 were significantly higher in DU145, PC3, and LNCaP compared with RWPE‐1. And DU‐145, PC‐3, and LNCaP are all derived from clinical metastatic lesions.^20^ Next, we aimed to elucidate the clinical implications of SALL4 in PCa.

### 3.2 High level of SALL4 correlates with unfavorable clinicopathological variables and poor prognosis in TCGA‐PRAD dataset

**FIGURE 2:**
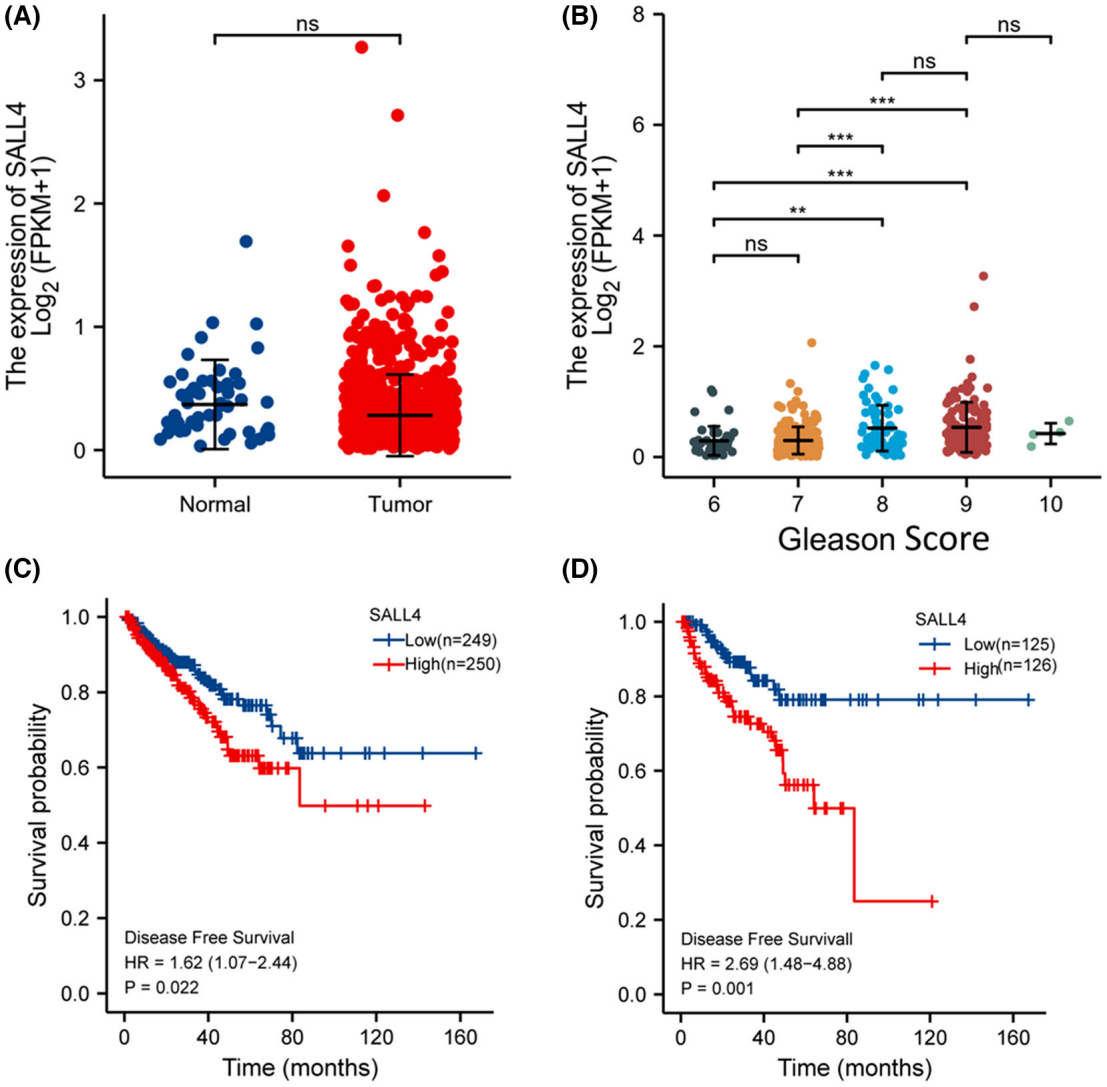
SALL4 upregulation in PCa patients is strongly associated with poor prognosis in the TCGA‐PRAD dataset. (A) The mRNA level of SALL4 in PCa tissues (*n* = 499) and nontumoral tissues (*n* = 52) was determined by analyzing RNA‐seq data from the TCGA‐PRAD dataset (*p* > 0.05). (B) SALL4 expression was higher in the high Gleason score group. **(**C,D**)** The Kaplan–Meier method was applied to show the disease‐free survival analysis of SALL4, and the different group cutoff (median or quartile) showed that the higher the SALL4 expression, the worse the prognosis.

In the TCGA‐PRAD dataset, we found no difference in mRNA levels of SALL4 in PCa samples (*n* = 499) and paracancerous nontumor tissues (*n* = 52), as almost all PCa patients in the dataset were earlier stage patients and only three patients with mPCa (Figure 2A, Table 1). As demonstrated in Figure 2A, SALL4 expression levels varied relatively widely in tumors. Moreover, in the TCGA‐PRAD dataset, we found that as the Gleason score increased, the patients' SALL4 expression also gradually increased (Figure 2B). Correlation analysis showed a significant association between increased SALL4 expression and pathological T stage (*p* < 0.001), pathological N stage (*p* < 0.001), and Gleason score (*p* < 0.001) (Table 1). Although there was no significant difference between SALL4 expression levels and M stage (*p* = 0.119) and overall survival (*p* = 0.106), the number of cases was too small to be convincing. Furthermore, a significant association between SALL4 expression and disease‐free survival was identified. PCa samples were classified as low‐ or high‐levels of SALL4 expression by median or quartiles. As SALL4 expression elevated, patients had a worse prognosis (Figure 2C, D). Altogether, these data indicate that SALL4 upregulation is markedly associated with unfavorable clinicopathological variables and poor prognosis of PCa.

**TABLE 1:** Correlation of SALL4 expression with clinicopathological status in TCGA‐PRAD dataset.

Characteristic	Low expression of SALL4 (*n* = 249)	High expression of SALL4 (*n* = 250)	p
Age, *n* (%)			0.573
≤ 65	180 (50.7%)	175 (49.3%)	
> 65	69 (47.9%)	75 (52.1%)	
Pathologic T stage, *n* (%)			< 0.001
T2	122(64.6%)	67 (35.4%)	
T3	121 (41.4%)	171 (58.6%)	
T4	3 (27.3%)	8 (72.7%)	
Pathologic N stage, *n* (%)			< 0.001
N0	181 (52.2%)	166 (47.8%)	
N1	20 (25.3%)	59 (74.7%)	
M stage, *n* (%)			0.119
M0	232 (51.0%)	223 (49.0%)	
M1	0 (0.0%)	3 (100%)	
Gleason score, *n* (%)			< 0.001
6	29 (63.0%)	17 (37.0%)	
7	151 (61.1%)	96 (38.9%)	
8	24 (37.5%)	40 (62.5%)	
9	44 (31.9%)	94 (68.1%)	
10	1 (25.0%)	3 (75.0%)	
OS event, n (%)			0.106
Alive	245 (50.3%)	242 (49.7%)	
Dead	2 (20.0%)	8 (80.0%)	

### 3.3 Expression of SALL4 in clinical PCa tissues and correlation with clinical features

**FIGURE 3:**
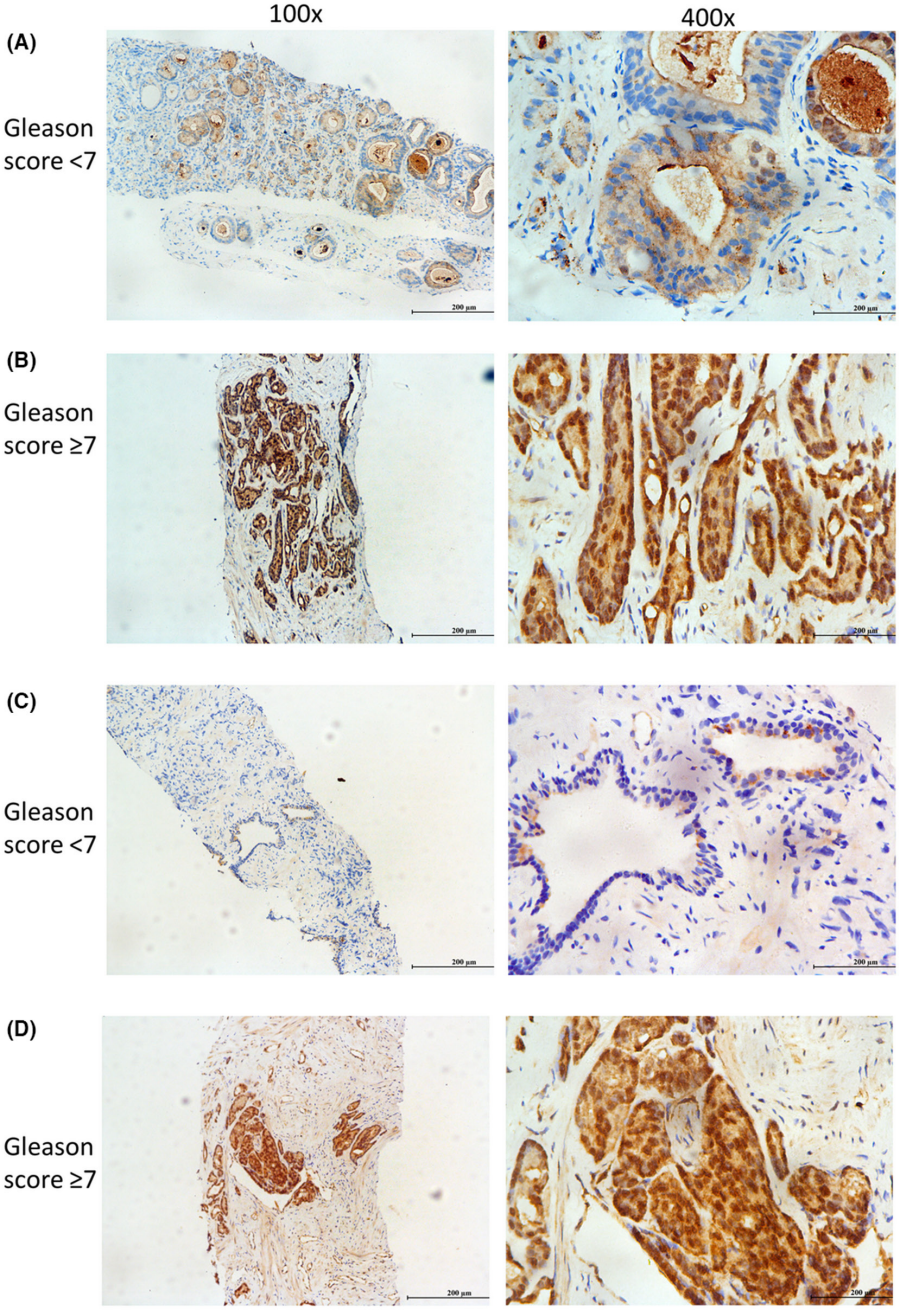
Immunohistochemical images showed the expression of SALL4 in prostate cancer tissues. (A, C) SALL4 expression in low Gleason score (<7) PCa tissues. (B, D) SALL4 expression in high Gleason score (≥7) PCa tissues.

To determine the expression of SALL4 in clinical samples, we examined the expression level of SALL4 in 68 PCa patients using immunohistochemical assays. As shown in Table 2, SALL4 expression levels were significantly related to clinical T stage (*p* < 0.01) and Gleason score (*p* < 0.05). Elevated expression of SALL4 was found in PCa tissues with high Gleason scores (≥7) compared with PCa tissues with low Gleason scores (<7) (Figure 3A–D). This was consistent with the findings of the TCGA‐PRAD dataset. In our cohort, there were no statistically significant differences between SALL4 expression levels and age (*p* = 0.636), PSA values (*p* = 0.314) and prostate volume (*p* = 0.739). Due to the small number of mPCa patients in 68 clinical PCa samples and the incomplete clinical information, we failed to compare SALL4 expression in mPCa and localized PCa. In summary, high expression of SALL4 was significantly associated with high clinical T stage and Gleason score in our clinical samples. Thus, we next explored the functional role of SALL4 in driving PCa metastasis.

**TABLE 2:** Correlation of SALL4 expression with clinical features in 68 PCa patients.

Characteristic	Total, *n*	SALL4 protein expression, *n*(%)			Z	p
		Low	Moderate	High		
Age, yrs					−0.473	0.636
≤65	35	2 (5.7)	7 (20.0)	26 (74.3)		
>65	33	1 (3.0)	6 (18.2)	26 (78.8)		
PSA, ng/mL					−1.006	0.314
≤10	32	3 (9.4)	6 (18.8)	23 (71.9)		
>10	36	0 (0)	7 (19.4)	29 (80.6)		
Clinical T stage					−2.775	<0.01
T1 + T2	30	2 (6.7)	10 (33.3)	18 (60.0)		
T3 + T4	38	1 (2.6)	3 (7.9)	34 (89.5)		
Gleason score					−2.084	<0.05
<7	21	3 (14.3)	5 (23.8)	13 (61.9)		
≥7	47	0 (0)	8 (17.0)	39 (83.0)		
Prostate volume (mL)					−0.333	0.739
≤25	23	1 (4.3)	5 (21.7)	17 (73.9)		
>25	45	2 (4.4)	8 (19.1)	35 (76.5)		

### 3.4 SALL4 correlates with PCa cells migration and tumor angiogenesis

**FIGURE 4:**
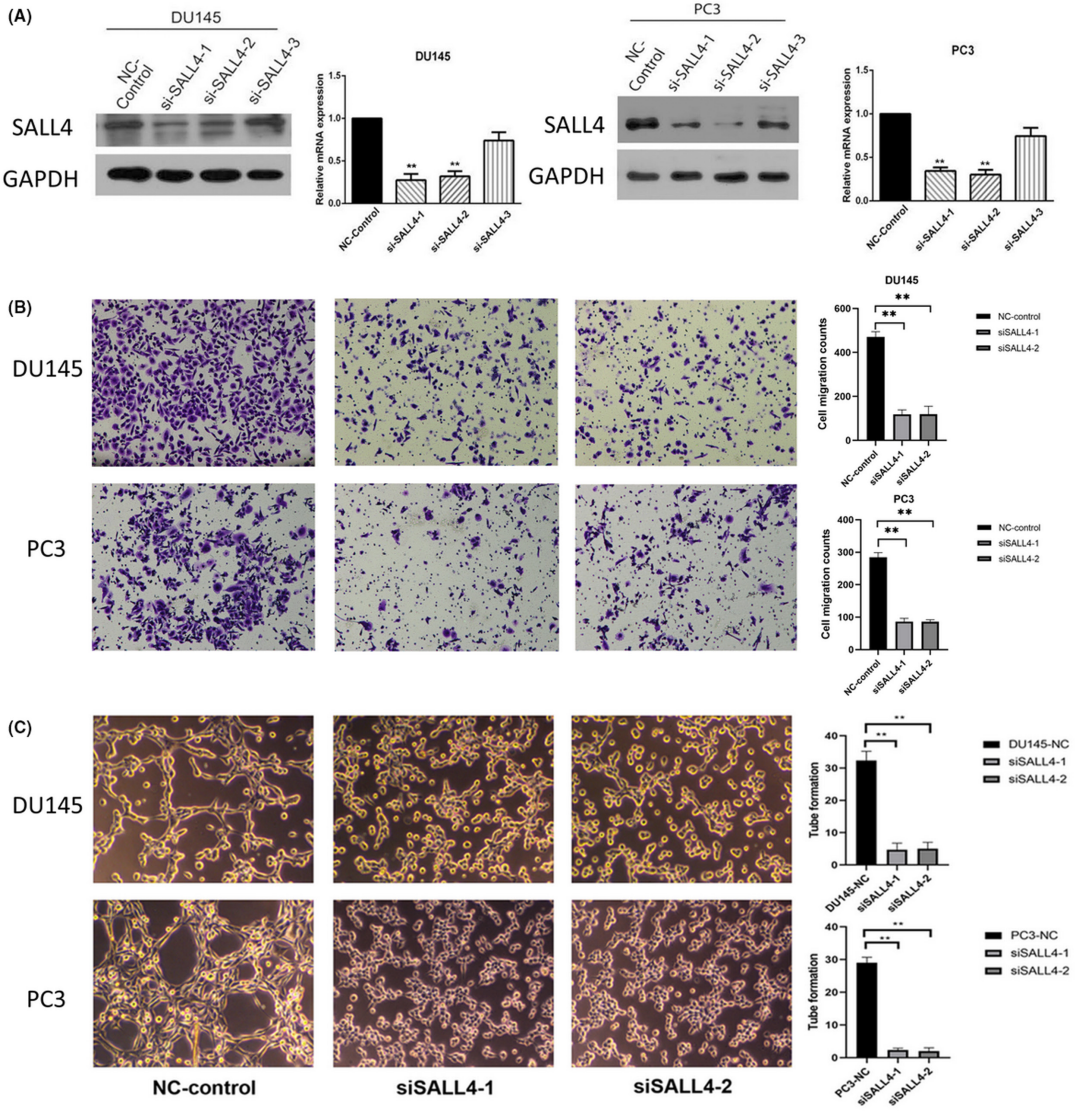
Knockdown of SALL4 impaired migration and tube formation ability of prostate cancer cells. (A) Validation of SALL4 knockdown in DU145 and PC3 cells by Western blot. (B) The transwell assay revealed that the migration ability of DU145 and PC3 cells was reduced after silencing SALL4. (C) The tube formation assay showed that tube formation significantly reduced after SALL4 knockdown. *p<0.05; **p<0.01.

To ascertain the function of SALL4 in PCa cells, we knocked down SALL4 in DU145 and PC3, which have higher metastatic capacity. SALL4 knockdown efficiency was verified by Western blot, and si‐SALL4‐1 and si‐SALL4‐2 were used for subsequent studies (Figure 4A). Then, we performed transwell assay to determine the impact of SALL4 on migration capacity in vitro. We found that the migration ability of both DU145 and PC3 was significantly reduced after knocking down SALL4 (*p* < 0.01) (Figure 4B). Considering that PCa metastasis is a multistep process in which tumor angiogenesis plays an important role, we then performed tube formation assay. We found that supernatants from SALL4 knocking down DU145 and PC3 can inhibit tube formation capacity of HUVECs (*p* < 0.01) (Figure 4C). Collectively, these in vitro studies demonstrate that SALL4 knockdown inhibits PCa metastasis.

### 3.5 SALL4 knockdown reduces proliferation and induces G1/S arrest in vitro

**FIGURE 5:**
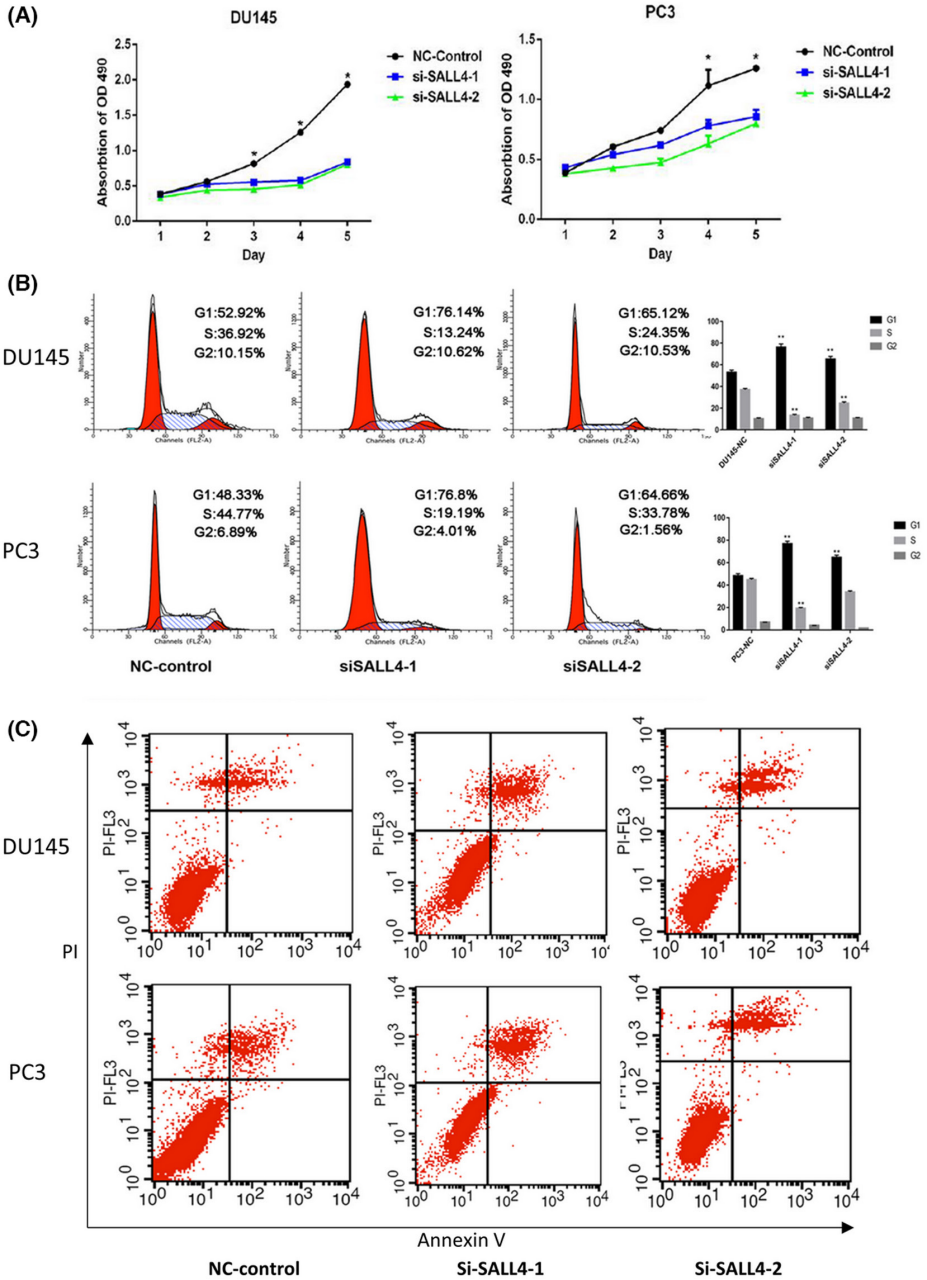
SALL4 knockdown reduced proliferation and induced G1/S arrest in vitro. (A) MTS assay showed the reduction in proliferation after silencing of SALL4 in DU145 and PC3 cells. (B) Cell cycle analysis by flow cytometry revealed that knockdown of SALL4 induced G1/S arrest in vitro. (C) SALL4 knockdown in DU145 and PC3 cells had no effect on apoptosis.

As mentioned previously, SALL4 is a transcription factor that is implicated in multiple biological processes. Cell cycle and cancer cell proliferation serve important roles in tumor progression, so we further explored the relationship between SALL4 and PCa cells proliferation. By MTS assay, we found that SALL4 was involved in the regulation of proliferation in PCa. The proliferative capacity of DU145 and PC3 cell lines was decreased after transfection with si‐SALL4‐1 and si‐SALL4‐2 siRNAs (Figure 5A). Cell cycle analysis showed that the proportion of DU145 and PC3 cells arrested in the G1 phase increased from 52.92% to 70.63% and 48.33% to 70.33%, respectively, after knocking down SALL4 (*p* < 0.01) (Figure 5B). Thus, we can know that SALL4 affects proliferation by inducing G1/S phase arrest.

Previous literature reported that SALL4 was associated with apoptosis, and then we further explored the correlation between SALL4 expression and apoptosis in PCa cells. Flow cytometry was applied to detect the proportion of apoptotic PCa cells after knockdown of SALL4. The results showed that apoptosis of DU145 and PC3 cells was not significant after knockdown of SALL4 (Figure 5C). Collectively, knockdown of SALL4 in PC3 and DU145 cells inhibited proliferation through G1/S phase arrest, but had no effect on apoptosis.

### 3.6 MAPK signaling pathway is involved in SALL4‐mediated PCa progression

**FIGURE 6:**
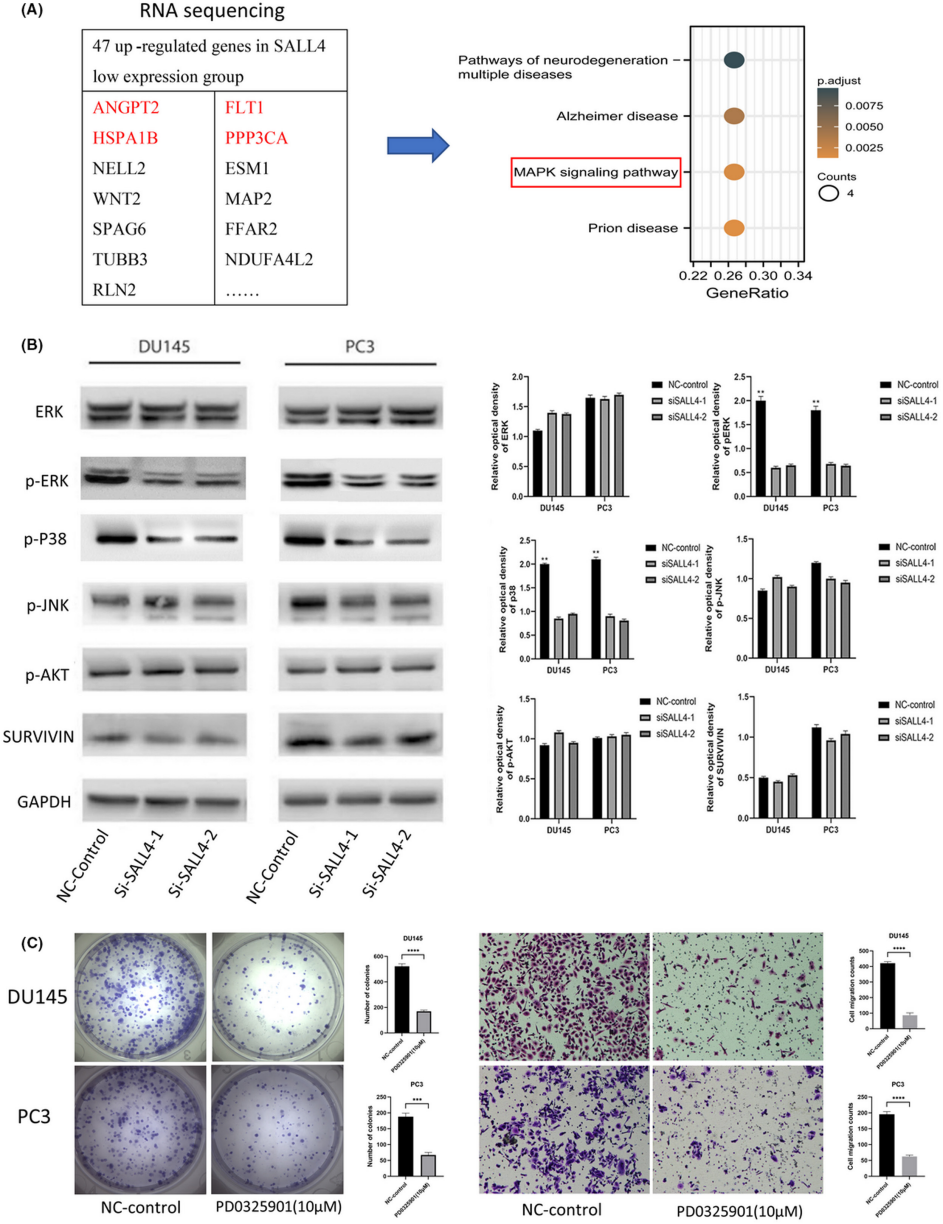
MAPK pathway is involved in SALL4‐mediated PCa progression. (A) In our RNA‐seq data, there were 47 significantly upregulated genes in the SALL4 low expression group, and enrichment analysis showed that the MAPK signaling pathway was enriched. (B) Phosphorylation of ERK and P38 was reduced in both DU145 and PC3 after knockdown of SALL4. (C) The proliferation and migration ability of DU145 and PC3 cells were significantly decreased after PD0325901 treatment.

Our previous in vitro experiments have suggested that SALL4 acts as an important role in promoting PCa progression. Then we focused on the mechanisms associated with SALL4 in driving PCa progression. Based on our RNA‐seq data, we divided the patients into SALL4 high expression and low expression groups according to their median expression. In the SALL4 low expression group, there were 47 upregulated genes (Table S5) with log2 fold change≥1 and adjust *p* value <0.05. Enrichment analysis of 47 upregulated genes showed that the mitogen‐activated protein kinase (MAPK) signaling pathway was enriched. Then, we reviewed the relevant literature and found that four upregulated genes, including ANGPT2, FLT1, HSPA1B, and PPP3CA, all negatively regulate the MAPK signaling pathway^21^, ^22^, ^23^, ^24^ (Figure 6A).

Therefore, we used Western blot to analyze the correlation between SALL4 and core proteins of MAPK pathway. We found that SALL4 knockdown induced a significant decrease in phosphorylation of ERK and P38, indicating the inactivation of the MAPK signaling pathway. We further investigated whether p‐AKT and p‐JNK are involved in SALL4‐mediated PCa progression and found that knockdown of SALL4 made no difference to phosphorylation of AKT and JNK (Figure 6B). We then treated DU145 and PC3 cells with PD0325901 and observed the same effects as SALL4 downregulation, that is, affecting prostate cancer cells proliferation and metastasis (Figure 6C). Combining these findings, our results demonstrate that ERK and P38 (MAPK signaling pathway) are involved in SALL4‐mediated PCa metastasis and progression.

## 4 DISCUSSION

Metastatic prostate cancer (mPCa) has a poor prognosis, and therefore the mechanisms underlying PCa metastasis need to be urgently understood. In this study, we integrated primary localized PCa tissues and primary mPCa tissues. Enrichment analysis of upregulated genes in our RNA‐seq and public dataset showed that the top‐ranked pathway was cell cycle. Accumulating evidences have shown that cell cycle‐related genes and pathways have an important impact on the occurrence and progression of cancer.^16^

Then by using our RNA‐seq and other datasets in public databases, we identified the cell cycle‐related gene SALL4 as an upregulated gene closely related to PCa progression. Both in the TCGA‐PRAD dataset and in our clinical samples, we found that high SALL4 expression and unfavorable outcome of PCa were associated. These findings prompted us to further investigate the effect of SALL4 on PCa progression.

As mentioned above, SALL4 is associated with many malignancies. In hepatocellular carcinoma patients, high levels of SALL4 correlated with poor prognosis. Similar results were found in leukemia, where SALL4 upregulation predicted advanced disease and poor prognosis.^25^, ^26^ In addition, SALL4 expression level was negatively correlated with postoperative prognosis of lung cancer patients.^27^ These results are in line with our findings. Our results demonstrated that high SALL4 expression was significantly associated with high pathological T stage, N stage, Gleason score, and poor disease‐free survival.

Related studies in other tumors have found that SALL4 is closely associated with tumor progression. In colorectal cancer, SALL4 could regulate differentiation and metastasis.^28^ The xenograft assay in immunodeficient mice showed that low expression levels of SALL4 dramatically reduced the tumorigenicity of NB4 cells.^29^ Similar studies have also shown an enrichment of proliferation‐ and metastasis‐related genes in SALL4 positive hepatocellular carcinomas. Moreover, knockdown of SALL4 induced apoptosis and cell cycle arrest in hematopoietic and leukemic cells by targeting Bmi‐1.^25^, ^30^ In our study we also found that knockdown of SALL4 impaired PCa progression. SALL4 knockdown inhibited proliferation, migration, and angiogenesis but not apoptosis in DU145 and PC3 cell lines. Consistently, SALL4 exerts its antiapoptotic function in a P53‐dependent manner,^31^ while both DU145 and PC3 are P53 mutated or deficient cells.^32^ Previous studies have found that SALL4 can promote angiogenesis,^33^ which was also in agreement with our results.

Recent studies have identified several downstream targets and signaling pathways of SALL4 in various types of cancers. According to the literature, overexpression of SALL4 can promote proliferation, invasion, and migration of cancer by activating Wnt/β‐catenin, PI3K/AKT and Notch signaling pathways as well as inhibiting the expression of the Bcl‐2 family, caspase‐related proteins and death receptors.^8^ In addition, SALL4 regulates gene expression through epigenetic mechanisms, which then influence tumor progression. Aberrant epigenetic modifications in the promoter region of the SALL4 were identified in patients with myelodysplastic syndrome (MDS).^25^ It is thus clear that SALL4, as a powerful transcription factor, can promote tumor metastasis in multiple ways. To further understand the mechanisms by which SALL4 promotes PCa progression, we explored possible downstream signaling pathways of SALL4 by enrichment analysis. The MAPK signaling pathway was then enriched and was further confirmed.

The MAPK pathway is among the most frequently affected signaling pathways in malignancies.^34^ Aberrant MAPK pathway plays a key role in the development and progression of human cancers and is involved in regulating cell growth, apoptosis, proliferation, differentiation, migration, immune, and stress responses.^35^, ^36^ The ERK signaling pathway is one of the four MAPK signaling pathways^37^ and is considered to be the best characterized MAPK signaling pathway associated with cell division, migration, and survival.^35^ P38 is another key protein in the MAPK pathway. Activation of p38 signaling plays an important role in the regulation of cell cycle, cell death, development, and tumorigenesis.^38^ Our results demonstrated that knockdown of SALL4 reduced the protein levels of p‐P38 and p‐ERK, while p‐JNK, p‐AKT, and SURVIVIN were not significantly changed. Therefore, it is reasonable to assume that knockdown of SALL4 affects the phosphorylation of ERK and P38 and inhibits the MAPK signaling pathway, thus slowing down the progression of PCa.

There have been many works related to the role of ERK and P38 in cancer progression. Many genes influence cancer development by regulating them. The most potential acridone, buxifoliadine E, inhibited the proliferation of four cancer cell lines, namely prostate cancer (LNCaP), neuroblastoma (SH SY5Y), hepatoblastoma (HepG2), and colorectal cancer (HT29), by inhibiting the ERK pathway.^39^ In prostate cancer, when ERK phosphorylation is attenuated, PC3 cell growth and angiogenesis can be inhibited.^40^ Elevated levels of phosphorylated ERK was also confirmed in castration‐resistant PCa compared to untreated primary PCa.^41^ For P38, our group has previously reported that it is related to proliferation, migration, and survival of LNCaP cells.^42^ Recently, Cheung et al. reported that inhibition of p38 MAPK activity decreased the proliferation and survival of CRPC cells in vitro and prolonged the survival time of tumor‐bearing mice.^43^ Similar findings were observed in other studies involving the P38 MAPK signaling pathway.^44^ Our current study also found that the proliferation and migration ability of DU145 and PC3 cells were significantly reduced after treatment with PD0325901. Collectively, combining the results of previous related studies and our findings, we can state that reduced phosphorylation of ERK and P38 proteins, that is, inactivation of MAPK signaling pathway, mediates a series of biological effects after SALL4 knockdown.

Our study also has some limitations. We elaborated the role of SALL4 in PCa progression, but did not explore its role in prostate carcinogenesis. Though we illustrated that high SALL4 expression was linked to unfavorable outcome, we failed to elucidate the association between SALL4 expression and overall survival because of the short follow‐up period both in TCGA and in our clinical data. Because ERK and P38 have been more studied and have clear functions in PCa development, we finally only elucidated that SALL4 can affect the phosphorylation of ERK and P38 without going further to do rescue experiments to confirm that whether SALL4 acts only through this pathway and whether there are other pathways involved in the regulation of cellular functions. More experiments are needed to verify these.

In summary, we integrated primary localized PCa tissues and primary mPCa tissues and performed RNA sequencing. Then, we identified SALL4 as an oncoprotein associated with PCa progression and found that SALL4 upregulation was significantly associated with unfavorable clinicopathological variables and poor prognosis of PCa. Furthermore, we confirmed that knockdown of SALL4 in DU145 and PC3 inhibited proliferation, migration, and angiogenesis. Such phenotypic changes in PCa cell lines after knockdown of SALL4 were found to be mainly caused by reduced ERK and P38 protein phosphorylation; that is, the inactivation of the MAPK cell signaling pathway. Collectively, SALL4 can be used as a potential prognostic marker and therapeutic target for PCa patients.

## AUTHOR CONTRIBUTIONS

**Jie zhou:** Data curation (equal); formal analysis (equal); methodology (equal); writing – original draft (equal). **Shengmeng Peng:** Data curation (equal); formal analysis (equal); methodology (equal); writing – original draft (equal). **Huiyang Fan:** Data curation (equal); formal analysis (equal); methodology (equal). **Jin Li:** Data curation (equal); formal analysis (equal); methodology (equal). **Zean Li:** Visualization (equal); writing – review and editing (equal). **Ganping Wang:** Visualization (equal); writing – review and editing (equal). **Lexiang Zeng:** Data curation (equal). **Zhenghui Guo:** Conceptualization (equal); funding acquisition (equal); supervision (equal). **Yiming Lai:** Conceptualization (equal); funding acquisition (equal); supervision (equal). **Hai Huang:** Conceptualization (equal); funding acquisition (equal); project administration (equal); supervision (equal).

## FUNDING INFORMATION

This work was supported by the National Natural Science Foundation of China (No: 81772733, 81972384), Guangdong scientific research projects (No: 2021A1515010223) to ZG. And also supported by the National Natural Science Foundation for Young Scientists of China (No: 81802527) and Beijing Bethune Charitable Foundation (No: mnzl202026) to YL. In addition, this work was supported by the National Natural Science Foundation of China (No: 81974395, No: 82173036); Guangdong Basic and Applied Basic Research Foundation (No: 2019A1515011437); International Science and technology cooperation project plan of Guangdong Province (No: 2021A0505030085); Sun Yat‐Sen University Clinical Research 5010 Program (No: 2019005); Sun Yat‐Sen Clinical Research Cultivating Program (No: 201702); Guangdong Province Key Laboratory of Malignant Tumor Epigenetics and Gene Regulation (No:2020B1212060018OF006); Guangdong Provincial Clinical Research Center for Urological Diseases (2020B1111170006) Beijing Bethune Charitable Foundation (mnzl202001) Guangzhou Science and Technology Key R&D Project (202206010117) to Hai Huang.

## CONFLICT OF INTEREST STATEMENT

The authors have no conflicts of interest to declare.

## ETHICS STATEMENT

The use of clinical specimens and pathological data in this study was approved by Ethics Committee of Sun Yat‐sen Memorial Hospital, Sun Yat‐sen University, Guangzhou, China, and informed consent was obtained from all patients. Its registration number was SYSEC‐KY‐KS‐2020‐201.

## Supporting information


Tables S1–S5

## Data Availability

The datasets generated and/or analyzed during the current study are available from the corresponding author on reasonable request.
