# Supplementary material for: SALL4 correlates with proliferation, metastasis, and poor prognosis in prostate cancer by affecting MAPK pathway
Source: Cancer Med. 2023 Apr 29;12(12):13471–85. doi: 10.1002/cam4.5998 (PMC10315722; doi:10.1002/cam4.5998)
Supplement: Supplementary file 1 — Tables S1–S5 [file CAM4-12-13471-s001.docx]

**Table S1** Patient characteristics for RNA-sequencing.

| Patient | Age | TNM stage | Gleason score | Metastases therapy |
| --- | --- | --- | --- | --- |
| Localized PCa 1 | 69 | T3bN0M0 | 4+4 | - |
| Localized PCa 2 | 69 | T3aN0M0 | 3+4 | - |
| Localized PCa 3 | 68 | T3bN0M0 | 4+4 | - |
| Localized PCa 4 | 57 | T3bN0M0 | 4+3 | - |
| Localized PCa 5 | 68 | T3aN0M0 | 4+5 | - |
| Localized PCa 6 | 80 | T3aN0M0 | 4+4 | - |
| Localized PCa 7 | 73 | T3aN0M0 | 4+3 | - |
| Metastatic PCa 1 | 65 | T4N0M1b | 4+5 | Zoledronic acid |
| Metastatic PCa 2 | 70 | T4N0M1b | 4+4 | Zoledronic acid & Surgery |
| Metastatic PCa 3 | 68 | T4N1M1b | 4+4 | Surgery |
| Metastatic PCa 4 | 74 | T4N0M1b | 4+5 | No |
| Metastatic PCa 5 | 73 | T4N1M1b | 4+5 | No |
| Metastatic PCa 6 | 74 | T4N1M1b | 4+4 | No |

**Table S2**

| **si-RNA** | **sequences** |
| --- | --- |
| si-RNA1 (sense) | 5’-CCCUGAAAUUGCAGCAGUUTT-3’ |
| si-RNA1 (antisense) | 5’-AACUGCUGCAAUUUCAGGGTT-3’ |
| si-RNA2 (sense) | 5’-CCAGGACAUAAGCUAUUUATT-3’ |
| si-RNA2 (antisense) | 5’-UAAAUAGCUUAUGUCCUGGTT-3’ |
| si-RNA3 (sense) | 5’-GACCGUUCCAGUGUAAGAUTT-3’ |
| si-RNA3 (antisense) | 5’-AUCUUACACUGGAACGGUCTT-3’ |

**Table S3**

| **qPCR primers** | **sequences** |
| --- | --- |
| GAPDH-F | AAATCCCATCACCATCTTCCAG |
| GAPDH-R | TGAGTCCTTCCACGATACCAAA |
| SALL4-F | CCCGGCAGTAAGGACTGTC |
| SALL4-R | TCTCTGTCTTTAGGTACACCACA |

**Table S4**

| **Antibody** | **Company** | **Cat No.** |
| --- | --- | --- |
| GAPDH | Proteintech | 10494-1-AP |
| SURVIVIN | Epitomics | 2463-1 |
| p-AKT | Cell signaling technology | 4060 |
| p-JNK | Cell signaling technology | 4668 |
| p-P38 | Cell signaling technology | 4511 |
| p-ERK | Cell signaling technology | 4370 |
| ERK | Cell signaling technology | 9102 |
| SALL4 | Abcam | ab29112 |

**Table S5**

| **In SALL4 low-expression group** | **47 up-regulated genes** | MBLAC1 C11orf98 BORCS7 MORN2 KIF20B GPR155 DPCD SMPDL3A UQCC2  PTPN13 ZCRB1 PCDH17 GPR4 NDUFA4L2 C5orf49 MAP9 NME5 PPP3CA HBEGF  RAMP1 FLT1 TUBB3 ANKRD18A  MAP2 CPE ANGPT2 HSPA1B FAM13C SERHL2 PHYHIPL LOC388780 DPYSL4 POTEE FFAR2 POTEJ ADAMTS4 SPAG6 HPGD RLN2 WNT2 CA8 ESM1 ZIC2 ZIC5 CHI3L2 NELL2 POTED |
| --- | --- | --- |
|  | **131 down-regulated genes** | TGM4 PADI3 FUT6 NXPE4 FUT3 CLCA1 IRX1 HBA2 SLITRK3 CASP14 HBB HBA1 HS3ST5  BMP3 BCHE GAS2L2 C22orf42 TNNT3 DCAF12L1 IFITM5 MSLN TBX18 PLP1 PSCA MUC16 GRIN3A ALAS2 ADGRG6 PCDH8  NEFM GAL3ST3 PRDM16 MUC2 ABCC3  BBOX1 CDH19 L1CAM FOXL2NB POF1B  KRT7 CCNI2 CHAD DUOX2 FAM110C  TG SOWAHA ANGPTL7 FCGBP CAPN8  MPZ CNMD ZFP92 PMP2 CAPN13 GPR17  CYP4X1 OBSCN PRSS12 FAM167A GABRE  NRXN1 NOX1 RASGEF1C GDF7 SHROOM1  SCNN1B ALS2CL FAT2 GRIN3B GREM1  FER1L5 SLITRK5 TLE6 DUOX1 PCDH20  NRXN3 FIBIN VTCN1 CPAMD8 SLC26A2  AMN WNT10A TMEM63C CALML6 ENTPD3  SALL4 HAP1 CADM3 TRIM46 NPW FUT2  ZNF185 TTC22 STX1B NGEF GDNF COL9A3  MST1R MYH7B PIANP ANO9 VWA7 IGSF9B  SYPL2 TRIM17 PGGHG ABCA9 DPP9-AS1  MN1 ZC3HAV1L MYO15B GDNF-AS1  RNF207 SYT15 NBEAL2 TMEM63A  CAMKK1 RAPGEFL1 ZNF296 FMN1  FLCN COL21A1 CAPN3 ITPR3 TMC6 SHANK3  SGSM1 SNED1 KIF26A ZNF841 SZT2 |
